# New platinum(II) complexes with benzo­thia­zole ligands

**DOI:** 10.1107/S2056989016002826

**Published:** 2016-02-24

**Authors:** José A. Carmona-Negrón, Mayra E. Cádiz, Curtis E. Moore, Arnold L. Rheingold, Enrique Meléndez

**Affiliations:** a University of Puerto Rico, Department of Chemistry, PO Box 9019, Mayaguez, PR 00681, Puerto Rico; b University of California-San Diego, Department of Chemistry, Urey Hall 5128, 9500 Gilman Drive, La Jolla, CA 92093-0358, USA

**Keywords:** crystal structure, cisplatin, platinum(II), benzo­thia­zole, anti­cancer

## Abstract

Four new platinum(II) complexes, namely tetra­ethyl­ammonium tri­bromido­(2-methyl-1,3-benzo­thia­zole-κ*N*)platinate(II), [NEt_4_][PtBr_3_(C_8_H_7_NS)] (**1**), tetra­ethyl­ammonium tri­bromido­(6-meth­oxy-2-methyl-1,3-benzo­thia­zole-κ*N*)platinate(II), [NEt_4_][PtBr_3_(C_9_H_9_NOS)] (**2**), tetra­ethyl­ammonium tri­bromido­(2,5,6-trimethyl-1,3-benzo­thia­zole-κ*N*)platinate(II), [NEt_4_][PtBr_3_(C_10_H_11_NS)] (**3**), and tetra­ethyl­ammonium tri­bromido­(2-methyl-5-nitro-1,3-benzo­thia­zole-κ*N*)platinate(II), [NEt_4_][PtBr_3_(C_8_H_6_N_2_O_2_S)] (**4**), have been synthesized and structurally characterized by single-crystal X-ray diffraction techniques. These species are precursors of compounds with potential application in cancer chemotherapy. All four platinum(II) complexes adopt the expected square-planar coordination geometry, and the benzo­thia­zole ligand is engaged in bonding to the metal atom through the imine N atom (Pt—N). The Pt—N bond lengths are normal: 2.035 (5), 2.025 (4), 2.027 (5) and 2.041 (4) Å for complexes **1**, **2**, **3** and **4**, respectively. The benzo­thia­zole ligands are positioned out of the square plane, with dihedral angles ranging from 76.4 (4) to 88.1 (4)°. The NEt_4_ cation in **3** is disordered with 0.57/0.43 occupancies.

## Abstract 2

Four new platinum(II) complexes, [NEt_4_][PtBr_3_(*L*)], containing benzo­thia­zole ligands have been structurally characterized by single-crystal X-ray diffraction techniques. All complexes adopt the expected square-planar coordination geometry, and the benzo­thia­zole is engaged in bonding to the metal atom through the imine N atom (Pt—N).

## Chemical context   

The synthesis of new platinum complexes as potential drugs for cancer is still of inter­est for medicinal chemists. The structural details of these complexes provide the opportunity to predict, to a certain extent, the potential biological activity of these species. In this regard, four new platinum(II) complexes with benzo­thia­zole ligands of general formula [PtBr_3_ *L*]^−^ have been synthesized according to the equation below and their structures characterized.[NEt_4_]_2_[Pt_2_Br_6_] + 2*L* → 2 [NEt_4_][PtBr_3_ *L*]*L* = 2-methyl-1,3-benzo­thia­zole (**1**), 6-meth­oxy-2-methyl-1,3-benzo­thia­zole (**2**), 2,5,6-trimethyl-1,3-benzo­thia­zole (**3**), and 2-methyl-5-nitro-1,3-benzo­thia­zole (**4**). All complexes showed the benzo­thia­zoles to coordinate the Pt^II^ atom through the imino nitro­gen atom. Also, the benzo­thia­zole is positioned out of the square plane with dihedral angles between 76.4 (4) and 88.1 (4)°, as previously reported in other platinum–benzo­thia­zole complexes. Given that benzo­thia­zoles have anti­cancer properties, these platinum complexes may have enhanced properties as a result of potential synergism between the ligand and Pt^II^. This deserves further studies as suggested by Noolvi *et al.* (2012 ▸)
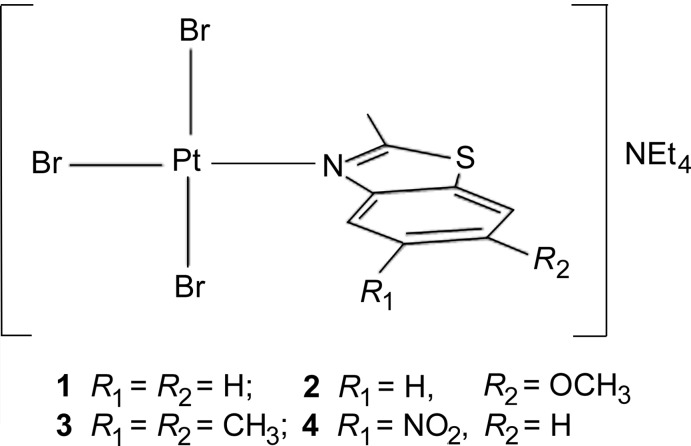
.

## Structural commentary   

To elucidate with certainty and accurately the platinum coordination patterns, the structural determination of the complexes was performed by single crystal X-ray diffraction technique. Table 1 ▸ contains selected bond lengths, dihedral angles and torsion angles. All of the title complexes adopt a square-planar coordination geometry about the Pt^II^ atom with a deviation of no more than 4° from ideal 180° and 90° angles. As reported previously, although not predicted using Pearson’s hard–soft acid base theory, the benzo­thia­zole is engaged in bonding to the metal through the imine nitro­gen (Pt—N) instead of Pt—S coordination (Muir *et al.*, 1987 ▸, 1988*a* ▸,*b* ▸, 1990 ▸; Gomez *et al.*, 1988 ▸; Lozano *et al.*, 1994 ▸). Also the benzo­thia­zole ligand is positioned out of the square plane as discussed below.

**Figure 1:**
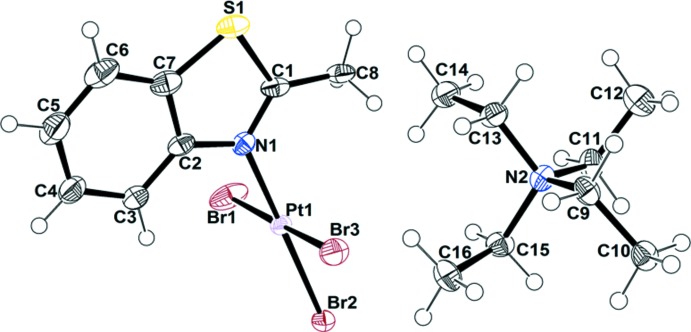
The mol­ecular structure of [NEt_4_][PtBr_3_(2-Me-benzo­thia­zole)] (**1**), with displacement ellipsoids drawn at the 50% probability level.

Figs. 1 ▸–4 ▸ ▸ ▸ show the mol­ecular structures of the four new complexes. [NEt_4_][PtBr_3_(2-Me-benzo­thia­zole)] (**1**) crystallizes in an ortho­rhom­bic unit cell with eight formula units. It is a square-planar complex with Pt—N and average Pt—Br bond lengths of 2.035 (5) and 2.433 (6) Å, respectively, which are within the expected range for Pt^II^ complexes. There is no *trans*-influence observed in the Pt—Br bond *trans* to the Pt—N bond. The benzo­thia­zole ligand is planar and the methyl group resides in the ligand plane. The dihedral angle between the PtBr_3_N unit and the benzo­thia­zole ring is 88.1 (4)°, similar to those observed in other Pt^II^–benzo­thia­zole complexes, as a result of reducing the steric strain between PtBr_3_ and the benzo­thia­zole ligand (Muir *et al.*, 1987 ▸, 1988*a* ▸,*b* ▸, 1990 ▸; Gomez *et al.*, 1988 ▸; Lozano *et al.*, 1994 ▸). Two types of N—C bonds are present, one long [N—C2 1.408 (7) Å] and one short [N—C1 1.309 (7) Å], indicating the presence of single- and double-bond character in the thia­zole ring. The angle at the S atom in the thia­zole ring is 90.3 (3)° suggesting it is using unhybridized *p* orbitals for bonding.

[NEt_4_][PtBr_3_(6-OMe-2-Me-benzo­thia­zole)] (**2**), [NEt_4_][PtBr_3_(2,5,6-Me-benzo­thia­zole)] (**3**) and [NEt_4_][PtBr_3_(5-NO_2_-2-Me-benzo­thia­zole)] (**4**) crystallize in the same type of unit cell and space group, monoclinic *P*2_1_/*n*, containing four formula units. The Pt—N and average Pt—Br bond lengths for **2**, **3**, and **4** are 2.025 (4)/2.430 (6) Å, 2.027 (5)/2.425 (6) Å and 2.041 (4)/2.431 (8) Å, respectively, which are within the expected range. The dihedral angle between PtBr_3_N and the benzo­thia­zole in **2** is 86.7 (3)° and the torsion angle between the aromatic ring and the OCH_3_ group is 11.9 (7)°^.^ The C—O (OCH_3_) bond length is 1.427 (7) Å, and the C—O—CH_3_ angle is 116.3 (5)°. In contrast to **1** and **2**, [NEt_4_][PtBr_3_(2,5,6-Me-benzo­thia­zole)] and [NEt_4_][PtBr_3_(5-NO_2_-2-Me-benzo­thia­zole)] have lower dihedral angles between the PtBr_3_N unit and the benzo­thia­zole ring, 78.6 (4) and 76.(4)°, respectively. The methyl groups on **3** and **4** are almost co-planar with the benzo­thia­zole plane with deviations ≤ 1.60° but in **4**, the NO_2_ group is out of the benzo­thia­zole plane with a torsion angle of 7.5 (7)°. The C—NO_2_ bond length is 1.476 (7) Å, and the O—N—O angle is 117.4 (5)°. The C—NO_2_ bond length and O—N—O angle in **4** are smaller than those observed in nitro­benzene [C—NO_2_ = 1.486 (2) Å and O—N—O = 123.9 (5)°], which suggests higher electron delocal­ization between the nitro group and the aromatic ring in **4** (Johnson, 2015 ▸). The angles at the S atom in **2**, **3** and **4** are also near 90°, suggesting the use of pure *p* orbitals for bonding.

## Supra­molecular features   

**Figure 5:**
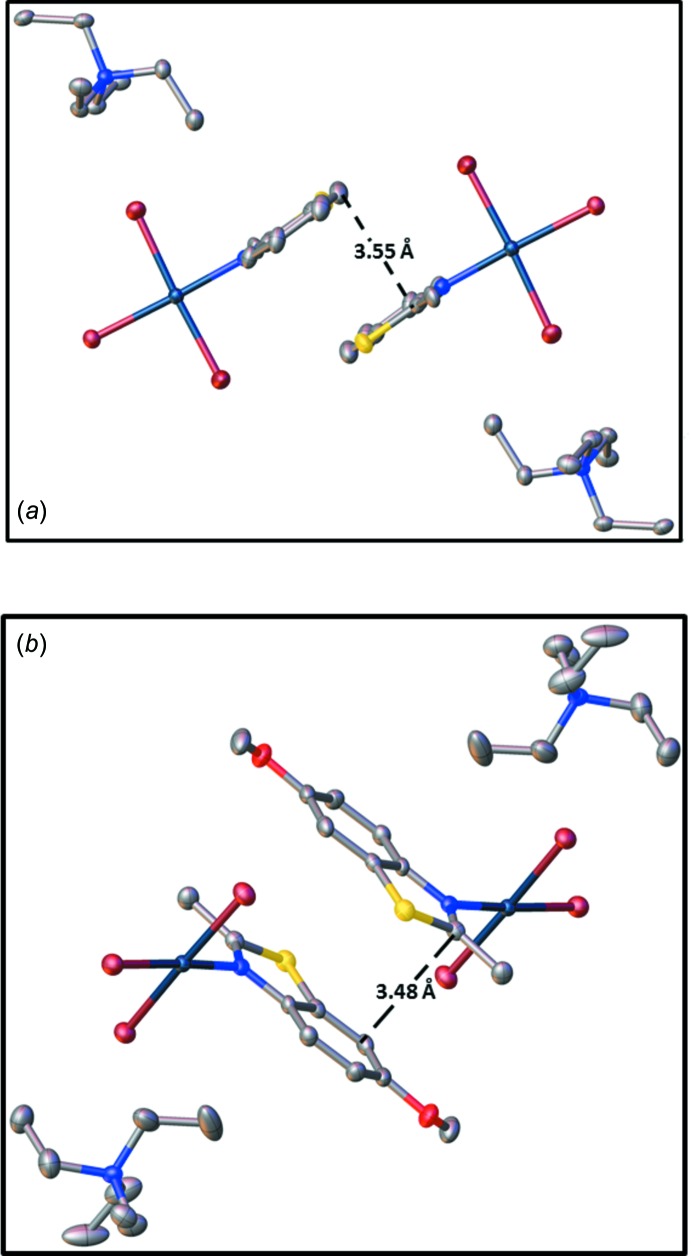
Details of the packing inter­actions in (*a*) [NEt_4_][PtBr_3_(2-Me-benzo­thia­zole)] and (*b*) [NEt_4_][PtBr_3_(6-Ome-2-Me-benzo­thia­zole)].

Analysis of the packing diagrams of all of the complexes showed their packings consist of [NEt_4_]^+^ cations and [PtBr_3_(*L*)]^−^ anions. The [NEt_4_][PtBr_3_(2-Me-benzo­thia­zole)] and [NEt_4_][PtBr_3_(6-OMe-2-Me-benzo­thia­zole)] complexes showed partial π-stacking between the phenyl and the thia­zole rings (Fig. 5 ▸).

## Synthesis and crystallization   

The parent complex [NEt_4_]_2_[Pt_2_Br_6_] was prepared as reported in the literature (Livingstone & Whitley, 1962 ▸). Ligands were purchased from Sigma–Aldrich and were used without further purification.

Acetone solutions of [NEt_4_]_2_[Pt_2_Br_6_] were prepared (0.075 g, 0.068 mmol) and the corresponding amount of ligand was added with stirring. For 2-methyl-1,3-benzo­thia­zole (99%) 18 μL (0.021 g, 0.14 mmol) were added; for 2-methyl-5-nitro-1,3-benzo­thia­zole (98%) (0.027 g, 0.14 mmol) were added, and for 2-methyl-6-meth­oxy-1,3-benzo­thia­zole (97%) (0.024 g, 0.14 mmol) were added. The reaction mixtures were stirred without heating until the volume reduced considerably; then the samples were placed in desiccators containing CaCl_2_ at room temperature to evaporate slowly, leading to the formation of X-ray quality single crystals. For the synthesis with 2,5,6-trimethyl-1,3-benzo­thia­zole (99%), the ligand (0.0227 g, 0.128 mmol) was added to 20 mL of an acetone solution (0.07515 g, 0.0677 mmol) of [NEt_4_]_2_[Pt_2_Br_6_] with stirring, and a portion of the reaction mixture was slowly evaporated at 277 K in a small beaker in a secondary container which also contained CaCl_2_ to form X-ray quality single crystals.

## Refinement   

Crystal data, data collection and structure refinement details are summarized in Table 2 ▸. H atoms were positioned in idealized locations: *d*(C—H) = 0.95 Å, *U*_iso_(H) = 1.2*U*_eq_(C); *d*(C—H_2_) = 0.99 Å, *U*_iso_(H) = 1.2*U*_eq_(C); *d*(C—H_3_) = 0.98 Å, *U*_iso_(H) = 1.5*U*_eq_(C). The NEt_4_ cation in **3** presented disorder with 0.57/0.43 occupancies.

## Supplementary Material

Crystal structure: contains datablock(s) 1, 2, 3, 4. DOI: 10.1107/S2056989016002826/bg2580sup1.cif

Structure factors: contains datablock(s) 1. DOI: 10.1107/S2056989016002826/bg25801sup2.hkl

Structure factors: contains datablock(s) 2. DOI: 10.1107/S2056989016002826/bg25802sup3.hkl

Structure factors: contains datablock(s) 3. DOI: 10.1107/S2056989016002826/bg25803sup4.hkl

Structure factors: contains datablock(s) 4. DOI: 10.1107/S2056989016002826/bg25804sup5.hkl

CCDC references: 1441324, 1441327, 1441326, 1441325

Additional supporting information:  crystallographic information; 3D view; checkCIF report

## Figures and Tables

**Figure 2:**
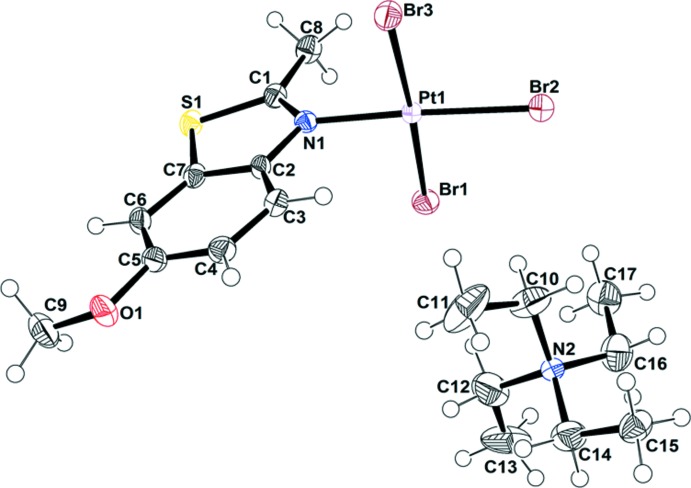
The mol­ecular structure of [NEt_4_][PtBr_3_(6-OMe-2-Me-benzo­thia­zole)] (**2**), with displacement ellipsoids drawn at the 50% probability level.

**Figure 3:**
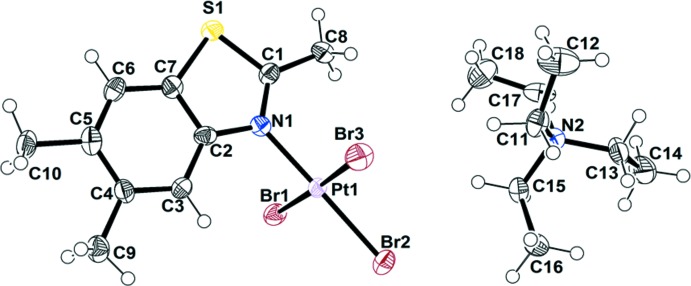
The mol­ecular structure of [NEt_4_][PtBr_3_(2,5,6-Me-benzo­thia­zole)] (**3**), with displacement ellipsoids drawn at the 50% probability level. The NEt_4_ cation in **3** presented disorder with 0.57/0.43 occupancies. Only the major fraction is shown for clarity.

**Figure 4:**
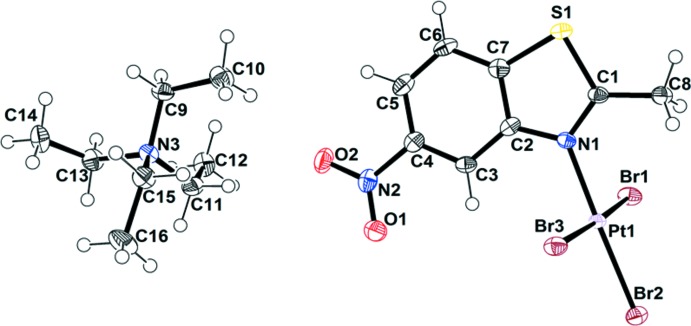
The mol­ecular structure of [NEt_4_][PtBr_3_(5-NO_2_-2-Me-benzo­thia­zole)] (**4**), with displacement ellipsoids drawn at the 50% probability level.

**Table 1:** Selected bond distances and angles (Å, °) The dihedral angle is between the Pt–Br_3_N unit and the benzo­thia­zole ring. The torsion angle is between the benzo­thia­zole ring and the *R* group.

	**1**	**2**	**3**	**4**
Pt—Br_average_	2.433 (6)	2.430 (6)	2.425 (6)	2.431 (8)
Pt—N	2.035 (5)	2.025 (4)	2.027 (5)	2.041 (4)
N1—C2	1.408 (7)	1.396 (6)	1.401 (8)	1.383 (6)
N1—C1	1.309 (7)	1.309 (6)	1.303 (8)	1.315 (6)
Pt—Br1	2.4375 (8)	2.4352 (5)	2.4309 (7)	2.4335 (6)
Pt—Br2	2.4349 (8)	2.4241 (7)	2.4198 (7)	2.4216 (5)
Pt—Br3	2.4268 (7)	2.4309 (5)	2.4240 (7)	2.4367 (5)
S—C7	1.744 (6)	1.743 (5)	1.739 (7)	1.738 (5)
S—C1	1.735 (6)	1.730 (5)	1.727 (6)	1.724 (5)
				
C1—N1—C2	113.0 (5)	112.6 (4)	112.3 (5)	111.9 (4)
C1—S—C7	90.3 (3)	89.9 (2)	89.8 (3)	90.0 (2)
N1—Pt—Br1	90.6 (1)	87.0 (1)	89.2 (1)	88.6 (1)
N1—Pt—Br3	86.4 (1)	89.3 (1)	88.5 (1)	89.3 (1)
N1—Pt—Br2	177.7 (1)	177.4 (1)	178.8 (1)	178.4 (1)
Br1—Pt—Br3	176.85 (2)	176.30 (2)	177.45 (3)	176.23 (2)
Br2—Pt—Br3	91.69 (2)	92.51 (2)	91.23 (2)	91.18 (2)
Br1—Pt—Br2	91.31 (2)	91.17 (2)	91.10 (2)	90.99 (2)
				
Dihedral angle	88.1 (4)	86.7 (3)	78.6 (4)	76.4 (4)
				
Torsion angle	0.72 (1) (CH_3_)	11.9 (7) (OCH_3_)	1.5 (5) (C_8_H_3_)	1.1 (5) (CH_3_)
			0.2 (6) (C_9_H_3_)	7.5 (7) (NO_2_)
			0.3 (6) (C_10_H_3_)	

**Table 2:** Experimental details

	(**1**)	(**2**)	(**3**)	(**4**)
Crystal data				
Chemical formula	(C_8_H_20_N)[PtBr_3_(C_8_H_7_NS)]	(C_8_H_20_N)[PtBr_3_(C_9_H_9_NOS)]	(C_8_H_20_N)[PtBr_3_(C_10_H_11_NS)]	(C_8_H_20_N)[PtBr_3_(C_8_H_6_N_2_O_2_S)]
*M*_r_	714.27	744.30	742.33	759.28
Crystal system, space group	Orthorhombic, *P* *b* *c* *a*	Monoclinic, *P*2_1_/*n*	Monoclinic, *P*2_1_/*n*	Monoclinic, *P*2_1_/*n*
Temperature (K)	100	100	100	100
*a*, *b*, *c* (Å)	12.114 (3), 10.656 (3), 34.043 (9)	7.7591 (2), 30.4214 (8), 9.6551 (3)	7.9742 (4), 30.2807 (14), 9.6427 (5)	8.1170 (3), 29.2717 (12), 9.5102 (4)
α, β, γ (°)	90, 90, 90	90, 94.539 (1), 90	90, 100.151 (3), 90	90, 100.720 (1), 90
*V* (Å^3^)	4394 (2)	2271.87 (11)	2291.9 (2)	2220.17 (15)
*Z*	8	4	4	4
Radiation type	Mo *K*α	Mo *K*α	Mo *K*α	Mo *K*α
μ (mm^−1^)	11.94	11.55	11.45	11.83
Crystal size (mm)	0.18 × 0.16 × 0.12	0.32 × 0.30 × 0.24	0.50 × 0.36 × 0.25	0.32 × 0.30 × 0.25

Data collection				
Diffractometer	Bruker APEXII CCD	Bruker APEXII CCD	Bruker APEXII CCD	Bruker APEXII CCD
Absorption correction	Multi-scan (*SADABS*; Bruker, 2014 ▸)	Multi-scan (*SADABS*; Bruker, 2014 ▸)	Multi-scan (*SADABS*; Bruker, 2014 ▸)	Multi-scan (*SADABS*; Bruker, 2014 ▸)
*T*_min_, *T*_max_	0.052, 0.093	0.056, 0.093	0.003, 0.028	0.020, 0.045
No. of measured, independent and observed [*I* > 2σ(*I*)] reflections	16951, 4418, 3675	12741, 4650, 4377	10729, 4692, 4120	15975, 4550, 4254
*R*_int_	0.047	0.017	0.048	0.028
(sin θ/λ)_max_ (Å^−1^)	0.623	0.626	0.627	0.627

Refinement				
*R*[*F* ^2^ > 2σ(*F* ^2^)], *wR*(*F* ^2^), *S*	0.031, 0.081, 1.03	0.027, 0.066, 1.08	0.039, 0.106, 1.05	0.029, 0.060, 1.18
No. of reflections	4418	4650	4692	4550
No. of parameters	213	232	266	240
H-atom treatment	H-atom parameters constrained	H-atom parameters constrained	H-atom parameters constrained	H-atom parameters constrained
Δρ_max_, Δρ_min_ (e Å^−3^)	2.38, −0.93	1.25, −1.36	1.88, −1.02	1.25, −1.37

Computer programs: *APEX2* and *SAINT* (Bruker, 2013 ▸), *SHELXT* (Sheldrick, 2015 ▸ *a*), *SIR2004* (Burla *et al.*, 2007 ▸), *SHELXL2014* (Sheldrick, 2015 ▸ *b*) and *OLEX2* (Dolomanov *et al.*, 2009 ▸).

## supplementary crystallographic information

### (1) Tetraethylammonium tribromido(2-methyl-1,3-benzothiazole-κN)platinate(II) Crystal data

**Table:**

(C_8_H_20_N)[PtBr_3_(C_8_H_7_NS)]	*D*_x_ = 2.159 Mg m^−^^3^
*M**_r_* = 714.27	Mo *K*α radiation, λ = 0.71073 Å
Orthorhombic, *P**b**c**a*	Cell parameters from 5330 reflections
*a* = 12.114 (3) Å	θ = 2.4–26.3°
*b* = 10.656 (3) Å	µ = 11.94 mm^−^^1^
*c* = 34.043 (9) Å	*T* = 100 K
*V* = 4394 (2) Å^3^	Block, bronze
*Z* = 8	0.18 × 0.16 × 0.12 mm
*F*(000) = 2688	

### (1) Tetraethylammonium tribromido(2-methyl-1,3-benzothiazole-κN)platinate(II) Data collection

**Table:**

Bruker APEXII CCD diffractometer	4418 independent reflections
Radiation source: Micro Focus Rotating Anode, Bruker TXS	3675 reflections with *I* > 2σ(*I*)
Double Bounce Multilayer Mirrors monochromator	*R*_int_ = 0.047
Detector resolution: 7.9 pixels mm^-1^	θ_max_ = 26.3°, θ_min_ = 2.1°
φ and ω scans	*h* = −14→15
Absorption correction: multi-scan (*SADABS*; Bruker, 2014)	*k* = −13→10
*T*_min_ = 0.052, *T*_max_ = 0.093	*l* = −32→42
16951 measured reflections	

### (1) Tetraethylammonium tribromido(2-methyl-1,3-benzothiazole-κN)platinate(II) Refinement

**Table:**

Refinement on *F*^2^	0 restraints
Least-squares matrix: full	Hydrogen site location: inferred from neighbouring sites
*R*[*F*^2^ > 2σ(*F*^2^)] = 0.031	H-atom parameters constrained
*wR*(*F*^2^) = 0.081	*w* = 1/[σ^2^(*F*_o_^2^) + (0.0352*P*)^2^ + 9.4131*P*] where *P* = (*F*_o_^2^ + 2*F*_c_^2^)/3
*S* = 1.03	(Δ/σ)_max_ = 0.003
4418 reflections	Δρ_max_ = 2.38 e Å^−^^3^
213 parameters	Δρ_min_ = −0.93 e Å^−^^3^

### (1) Tetraethylammonium tribromido(2-methyl-1,3-benzothiazole-κN)platinate(II) Special details

**Table:**

Geometry. All e.s.d.'s (except the e.s.d. in the dihedral angle between two l.s. planes) are estimated using the full covariance matrix. The cell e.s.d.'s are taken into account individually in the estimation of e.s.d.'s in distances, angles and torsion angles; correlations between e.s.d.'s in cell parameters are only used when they are defined by crystal symmetry. An approximate (isotropic) treatment of cell e.s.d.'s is used for estimating e.s.d.'s involving l.s. planes.

### (1) Tetraethylammonium tribromido(2-methyl-1,3-benzothiazole-κN)platinate(II) Fractional atomic coordinates and isotropic or equivalent isotropic displacement parameters (Å^2^)

**Table:**

	*x*	*y*	*z*	*U*_iso_*/*U*_eq_	
N2	1.0097 (4)	0.4493 (4)	0.31862 (12)	0.0213 (9)	
C9	1.1117 (4)	0.3738 (5)	0.30557 (16)	0.0242 (12)	
H9A	1.1049	0.2872	0.3158	0.029*	
H9B	1.1781	0.4117	0.3177	0.029*	
C10	1.1289 (4)	0.3677 (5)	0.26030 (15)	0.0251 (12)	
H10A	1.0624	0.3336	0.2479	0.038*	
H10B	1.1920	0.3134	0.2544	0.038*	
H10C	1.1431	0.4523	0.2502	0.038*	
C11	1.0130 (5)	0.5845 (5)	0.30315 (17)	0.0259 (12)	
H11A	1.0120	0.5820	0.2741	0.031*	
H11B	0.9452	0.6282	0.3119	0.031*	
C12	1.1120 (5)	0.6608 (5)	0.31618 (18)	0.0339 (14)	
H12A	1.1104	0.7430	0.3033	0.051*	
H12B	1.1799	0.6166	0.3089	0.051*	
H12C	1.1097	0.6720	0.3447	0.051*	
C13	1.0124 (5)	0.4436 (6)	0.36271 (15)	0.0259 (12)	
H13A	1.0827	0.4809	0.3719	0.031*	
H13B	1.0121	0.3543	0.3708	0.031*	
C14	0.9162 (5)	0.5110 (5)	0.38363 (18)	0.0305 (13)	
H14A	0.9172	0.6003	0.3768	0.046*	
H14B	0.9243	0.5017	0.4121	0.046*	
H14C	0.8460	0.4739	0.3753	0.046*	
C15	0.9038 (4)	0.3934 (5)	0.30181 (16)	0.0248 (12)	
H15A	0.8405	0.4417	0.3122	0.030*	
H15B	0.9048	0.4048	0.2730	0.030*	
C16	0.8844 (4)	0.2555 (5)	0.31049 (17)	0.0303 (13)	
H16A	0.9447	0.2056	0.2993	0.045*	
H16B	0.8142	0.2291	0.2988	0.045*	
H16C	0.8819	0.2426	0.3390	0.045*	
Pt1	0.47417 (2)	0.49411 (2)	0.37148 (2)	0.01931 (8)	
Br1	0.31934 (5)	0.60895 (7)	0.39974 (2)	0.04565 (19)	
Br2	0.39703 (4)	0.51437 (5)	0.30574 (2)	0.02401 (13)	
Br3	0.63067 (4)	0.37553 (6)	0.34693 (2)	0.03153 (15)	
S1	0.67084 (13)	0.48262 (14)	0.48520 (4)	0.0321 (3)	
N1	0.5409 (4)	0.4702 (4)	0.42583 (13)	0.0221 (10)	
C1	0.6192 (4)	0.5394 (5)	0.44109 (15)	0.0247 (12)	
C2	0.5158 (4)	0.3638 (5)	0.44867 (15)	0.0246 (12)	
C3	0.4356 (5)	0.2744 (5)	0.44047 (16)	0.0290 (12)	
H3	0.3925	0.2796	0.4172	0.035*	
C4	0.4200 (6)	0.1769 (6)	0.46718 (16)	0.0351 (14)	
H4	0.3642	0.1162	0.4624	0.042*	
C5	0.4853 (5)	0.1671 (7)	0.50112 (16)	0.0398 (16)	
H5	0.4740	0.0987	0.5186	0.048*	
C6	0.5666 (6)	0.2558 (6)	0.50970 (17)	0.0379 (15)	
H6	0.6106	0.2495	0.5327	0.045*	
C7	0.5807 (5)	0.3552 (5)	0.48283 (16)	0.0315 (13)	
C8	0.6643 (5)	0.6567 (5)	0.42301 (16)	0.0290 (13)	
H8A	0.7204	0.6929	0.4404	0.044*	
H8B	0.6043	0.7173	0.4193	0.044*	
H8C	0.6976	0.6368	0.3975	0.044*	

### (1) Tetraethylammonium tribromido(2-methyl-1,3-benzothiazole-κN)platinate(II) Atomic displacement parameters (Å^2^)

**Table:**

	*U*^11^	*U*^22^	*U*^33^	*U*^12^	*U*^13^	*U*^23^
N2	0.022 (2)	0.022 (2)	0.020 (2)	0.0039 (19)	0.0002 (18)	0.0024 (19)
C9	0.016 (3)	0.024 (3)	0.032 (3)	0.001 (2)	0.000 (2)	0.000 (2)
C10	0.019 (3)	0.025 (3)	0.032 (3)	0.003 (2)	0.004 (2)	−0.003 (2)
C11	0.026 (3)	0.021 (3)	0.031 (3)	0.002 (2)	0.004 (2)	0.006 (2)
C12	0.031 (3)	0.027 (3)	0.044 (4)	0.000 (3)	−0.004 (3)	0.002 (3)
C13	0.030 (3)	0.028 (3)	0.020 (3)	0.000 (3)	−0.001 (2)	−0.001 (2)
C14	0.030 (3)	0.037 (3)	0.025 (3)	−0.001 (3)	0.000 (2)	−0.003 (2)
C15	0.018 (3)	0.032 (3)	0.025 (3)	0.003 (2)	−0.005 (2)	−0.002 (2)
C16	0.021 (3)	0.031 (3)	0.039 (3)	−0.003 (2)	−0.001 (2)	0.002 (3)
Pt1	0.01841 (13)	0.02210 (12)	0.01742 (13)	0.00011 (8)	−0.00120 (7)	−0.00100 (8)
Br1	0.0399 (4)	0.0675 (5)	0.0296 (3)	0.0248 (3)	−0.0059 (3)	−0.0135 (3)
Br2	0.0241 (3)	0.0255 (3)	0.0224 (3)	−0.0012 (2)	−0.0022 (2)	−0.0005 (2)
Br3	0.0235 (3)	0.0411 (3)	0.0300 (3)	0.0027 (3)	0.0004 (2)	−0.0050 (3)
S1	0.0337 (8)	0.0381 (8)	0.0245 (7)	0.0055 (7)	−0.0105 (6)	−0.0028 (6)
N1	0.022 (2)	0.025 (2)	0.019 (2)	0.0030 (19)	0.0017 (18)	0.0000 (19)
C1	0.022 (3)	0.031 (3)	0.021 (3)	0.006 (2)	−0.004 (2)	−0.009 (2)
C2	0.030 (3)	0.026 (3)	0.018 (3)	0.005 (2)	0.001 (2)	−0.005 (2)
C3	0.033 (3)	0.032 (3)	0.021 (3)	−0.003 (3)	0.004 (2)	−0.005 (2)
C4	0.051 (4)	0.032 (3)	0.022 (3)	−0.004 (3)	0.006 (3)	−0.002 (2)
C5	0.062 (4)	0.032 (4)	0.026 (3)	0.003 (3)	0.005 (3)	0.003 (2)
C6	0.054 (4)	0.035 (3)	0.025 (3)	0.011 (3)	−0.004 (3)	0.001 (3)
C7	0.036 (3)	0.033 (3)	0.025 (3)	0.006 (3)	0.002 (3)	−0.010 (2)
C8	0.034 (3)	0.032 (3)	0.021 (3)	−0.001 (3)	−0.010 (2)	−0.004 (2)

### (1) Tetraethylammonium tribromido(2-methyl-1,3-benzothiazole-κN)platinate(II) Geometric parameters (Å, º)

**Table:**

N2—C9	1.540 (6)	C16—H16A	0.9800
N2—C11	1.534 (7)	C16—H16B	0.9800
N2—C13	1.503 (7)	C16—H16C	0.9800
N2—C15	1.526 (7)	Pt1—Br1	2.4375 (8)
C9—H9A	0.9900	Pt1—Br2	2.4349 (8)
C9—H9B	0.9900	Pt1—Br3	2.4268 (7)
C9—C10	1.557 (7)	Pt1—N1	2.035 (5)
C10—H10A	0.9800	S1—C1	1.735 (6)
C10—H10B	0.9800	S1—C7	1.744 (6)
C10—H10C	0.9800	N1—C1	1.309 (7)
C11—H11A	0.9900	N1—C2	1.408 (7)
C11—H11B	0.9900	C1—C8	1.497 (8)
C11—C12	1.515 (8)	C2—C3	1.389 (8)
C12—H12A	0.9800	C2—C7	1.407 (8)
C12—H12B	0.9800	C3—H3	0.9500
C12—H12C	0.9800	C3—C4	1.393 (8)
C13—H13A	0.9900	C4—H4	0.9500
C13—H13B	0.9900	C4—C5	1.405 (8)
C13—C14	1.543 (8)	C5—H5	0.9500
C14—H14A	0.9800	C5—C6	1.396 (9)
C14—H14B	0.9800	C6—H6	0.9500
C14—H14C	0.9800	C6—C7	1.410 (8)
C15—H15A	0.9900	C8—H8A	0.9800
C15—H15B	0.9900	C8—H8B	0.9800
C15—C16	1.517 (8)	C8—H8C	0.9800
			
C11—N2—C9	111.8 (4)	C16—C15—H15A	108.3
C13—N2—C9	104.5 (4)	C16—C15—H15B	108.3
C13—N2—C11	112.4 (4)	C15—C16—H16A	109.5
C13—N2—C15	112.1 (4)	C15—C16—H16B	109.5
C15—N2—C9	111.2 (4)	C15—C16—H16C	109.5
C15—N2—C11	105.1 (4)	H16A—C16—H16B	109.5
N2—C9—H9A	108.6	H16A—C16—H16C	109.5
N2—C9—H9B	108.6	H16B—C16—H16C	109.5
N2—C9—C10	114.5 (4)	Br2—Pt1—Br1	91.31 (2)
H9A—C9—H9B	107.6	Br3—Pt1—Br1	176.85 (2)
C10—C9—H9A	108.6	Br3—Pt1—Br2	91.69 (2)
C10—C9—H9B	108.6	N1—Pt1—Br1	90.56 (12)
C9—C10—H10A	109.5	N1—Pt1—Br2	177.68 (13)
C9—C10—H10B	109.5	N1—Pt1—Br3	86.41 (12)
C9—C10—H10C	109.5	C1—S1—C7	90.3 (3)
H10A—C10—H10B	109.5	C1—N1—Pt1	125.3 (4)
H10A—C10—H10C	109.5	C1—N1—C2	113.0 (5)
H10B—C10—H10C	109.5	C2—N1—Pt1	121.2 (4)
N2—C11—H11A	108.5	N1—C1—S1	114.1 (4)
N2—C11—H11B	108.5	N1—C1—C8	124.9 (5)
H11A—C11—H11B	107.5	C8—C1—S1	121.0 (4)
C12—C11—N2	115.1 (5)	C3—C2—N1	126.3 (5)
C12—C11—H11A	108.5	C3—C2—C7	120.8 (5)
C12—C11—H11B	108.5	C7—C2—N1	112.8 (5)
C11—C12—H12A	109.5	C2—C3—H3	120.8
C11—C12—H12B	109.5	C2—C3—C4	118.4 (5)
C11—C12—H12C	109.5	C4—C3—H3	120.8
H12A—C12—H12B	109.5	C3—C4—H4	119.5
H12A—C12—H12C	109.5	C3—C4—C5	121.0 (6)
H12B—C12—H12C	109.5	C5—C4—H4	119.5
N2—C13—H13A	108.5	C4—C5—H5	119.4
N2—C13—H13B	108.5	C6—C5—C4	121.3 (6)
N2—C13—C14	115.2 (5)	C6—C5—H5	119.4
H13A—C13—H13B	107.5	C5—C6—H6	121.4
C14—C13—H13A	108.5	C5—C6—C7	117.3 (6)
C14—C13—H13B	108.5	C7—C6—H6	121.4
C13—C14—H14A	109.5	C2—C7—S1	109.7 (4)
C13—C14—H14B	109.5	C2—C7—C6	121.2 (6)
C13—C14—H14C	109.5	C6—C7—S1	129.1 (5)
H14A—C14—H14B	109.5	C1—C8—H8A	109.5
H14A—C14—H14C	109.5	C1—C8—H8B	109.5
H14B—C14—H14C	109.5	C1—C8—H8C	109.5
N2—C15—H15A	108.3	H8A—C8—H8B	109.5
N2—C15—H15B	108.3	H8A—C8—H8C	109.5
H15A—C15—H15B	107.4	H8B—C8—H8C	109.5
C16—C15—N2	115.8 (4)		

### (2) Tetraethylammonium tribromido(6-methoxy-2-methyl-1,3-benzothiazole-κN)platinate(II) Crystal data

**Table:**

(C_8_H_20_N)[PtBr_3_(C_9_H_9_NOS)]	*F*(000) = 1408
*M**_r_* = 744.30	*D*_x_ = 2.176 Mg m^−^^3^
Monoclinic, *P*2_1_/*n*	Mo *K*α radiation, λ = 0.71073 Å
*a* = 7.7591 (2) Å	Cell parameters from 7838 reflections
*b* = 30.4214 (8) Å	θ = 2.2–26.4°
*c* = 9.6551 (3) Å	µ = 11.55 mm^−^^1^
β = 94.539 (1)°	*T* = 100 K
*V* = 2271.87 (11) Å^3^	Block, bronze
*Z* = 4	0.32 × 0.3 × 0.24 mm

### (2) Tetraethylammonium tribromido(6-methoxy-2-methyl-1,3-benzothiazole-κN)platinate(II) Data collection

**Table:**

Bruker APEXII CCD diffractometer	4650 independent reflections
Radiation source: Micro Focus Rotating Anode, Bruker TXS	4377 reflections with *I* > 2σ(*I*)
Double Bounce Multilayer Mirrors monochromator	*R*_int_ = 0.017
Detector resolution: 7.9 pixels mm^-1^	θ_max_ = 26.4°, θ_min_ = 2.2°
φ and ω scans	*h* = −9→9
Absorption correction: multi-scan (*SADABS*; Bruker, 2014)	*k* = −32→38
*T*_min_ = 0.056, *T*_max_ = 0.093	*l* = −12→7
12741 measured reflections	

### (2) Tetraethylammonium tribromido(6-methoxy-2-methyl-1,3-benzothiazole-κN)platinate(II) Refinement

**Table:**

Refinement on *F*^2^	0 restraints
Least-squares matrix: full	Hydrogen site location: inferred from neighbouring sites
*R*[*F*^2^ > 2σ(*F*^2^)] = 0.027	H-atom parameters constrained
*wR*(*F*^2^) = 0.066	*w* = 1/[σ^2^(*F*_o_^2^) + (0.0227*P*)^2^ + 15.6321*P*] where *P* = (*F*_o_^2^ + 2*F*_c_^2^)/3
*S* = 1.08	(Δ/σ)_max_ = 0.001
4650 reflections	Δρ_max_ = 1.25 e Å^−^^3^
232 parameters	Δρ_min_ = −1.36 e Å^−^^3^

### (2) Tetraethylammonium tribromido(6-methoxy-2-methyl-1,3-benzothiazole-κN)platinate(II) Special details

**Table:**

Geometry. All e.s.d.'s (except the e.s.d. in the dihedral angle between two l.s. planes) are estimated using the full covariance matrix. The cell e.s.d.'s are taken into account individually in the estimation of e.s.d.'s in distances, angles and torsion angles; correlations between e.s.d.'s in cell parameters are only used when they are defined by crystal symmetry. An approximate (isotropic) treatment of cell e.s.d.'s is used for estimating e.s.d.'s involving l.s. planes.

### (2) Tetraethylammonium tribromido(6-methoxy-2-methyl-1,3-benzothiazole-κN)platinate(II) Fractional atomic coordinates and isotropic or equivalent isotropic displacement parameters (Å^2^)

**Table:**

	*x*	*y*	*z*	*U*_iso_*/*U*_eq_	
N2	0.4744 (6)	0.68841 (14)	1.0072 (4)	0.0224 (9)	
C10	0.4365 (10)	0.6636 (2)	0.8727 (7)	0.0442 (16)	
H10A	0.3248	0.6744	0.8289	0.053*	
H10B	0.5266	0.6715	0.8099	0.053*	
C11	0.4277 (12)	0.6153 (2)	0.8795 (10)	0.065 (3)	
H11A	0.5326	0.6039	0.9305	0.097*	
H11B	0.4183	0.6032	0.7851	0.097*	
H11C	0.3263	0.6065	0.9274	0.097*	
C12	0.6550 (9)	0.6759 (3)	1.0680 (8)	0.0529 (19)	
H12A	0.6514	0.6451	1.1004	0.064*	
H12B	0.7336	0.6769	0.9921	0.064*	
C13	0.7308 (9)	0.7028 (3)	1.1828 (7)	0.061 (2)	
H13A	0.6425	0.7094	1.2470	0.092*	
H13B	0.7743	0.7304	1.1462	0.092*	
H13C	0.8264	0.6868	1.2322	0.092*	
C14	0.3484 (8)	0.6760 (2)	1.1136 (7)	0.0403 (15)	
H14A	0.3582	0.6440	1.1319	0.048*	
H14B	0.3825	0.6915	1.2018	0.048*	
C15	0.1616 (7)	0.6868 (2)	1.0702 (6)	0.0338 (13)	
H15A	0.0883	0.6767	1.1421	0.051*	
H15B	0.1267	0.6719	0.9824	0.051*	
H15C	0.1488	0.7186	1.0581	0.051*	
C16	0.4651 (9)	0.7366 (2)	0.9800 (8)	0.0426 (15)	
H16A	0.4764	0.7522	1.0703	0.051*	
H16B	0.3493	0.7435	0.9349	0.051*	
C17	0.6004 (9)	0.7545 (2)	0.8898 (7)	0.0425 (15)	
H17A	0.7160	0.7485	0.9340	0.064*	
H17B	0.5849	0.7864	0.8787	0.064*	
H17C	0.5876	0.7404	0.7984	0.064*	
Pt1	0.73847 (2)	0.62959 (2)	0.53521 (2)	0.01668 (6)	
Br1	0.90363 (7)	0.66953 (2)	0.71969 (5)	0.02744 (12)	
Br2	0.52471 (7)	0.68778 (2)	0.51179 (5)	0.02766 (12)	
Br3	0.58868 (7)	0.58627 (2)	0.35144 (6)	0.02867 (12)	
S1	1.20643 (16)	0.54060 (4)	0.58464 (13)	0.0233 (3)	
O1	0.8812 (6)	0.43871 (13)	0.9169 (4)	0.0337 (9)	
N1	0.9166 (5)	0.58111 (13)	0.5642 (4)	0.0184 (8)	
C1	1.0717 (6)	0.58239 (17)	0.5202 (5)	0.0213 (10)	
C2	0.8935 (6)	0.54544 (15)	0.6519 (5)	0.0191 (10)	
C3	0.7394 (7)	0.53338 (17)	0.7090 (5)	0.0222 (10)	
H3	0.6361	0.5497	0.6889	0.027*	
C4	0.7421 (7)	0.49725 (17)	0.7951 (5)	0.0255 (11)	
H4	0.6390	0.4885	0.8344	0.031*	
C5	0.8937 (8)	0.47324 (17)	0.8256 (5)	0.0262 (11)	
C6	1.0457 (7)	0.48345 (17)	0.7664 (5)	0.0250 (11)	
H6	1.1476	0.4664	0.7841	0.030*	
C7	1.0411 (6)	0.52035 (16)	0.6788 (5)	0.0204 (10)	
C8	1.1316 (7)	0.61649 (18)	0.4252 (6)	0.0277 (11)	
H8A	1.1838	0.6409	0.4799	0.042*	
H8B	1.0329	0.6273	0.3652	0.042*	
H8C	1.2175	0.6038	0.3678	0.042*	
C9	1.0403 (9)	0.4191 (2)	0.9692 (7)	0.0393 (15)	
H9A	1.0908	0.4030	0.8943	0.059*	
H9B	1.0187	0.3987	1.0445	0.059*	
H9C	1.1206	0.4420	1.0046	0.059*	

### (2) Tetraethylammonium tribromido(6-methoxy-2-methyl-1,3-benzothiazole-κN)platinate(II) Atomic displacement parameters (Å^2^)

**Table:**

	*U*^11^	*U*^22^	*U*^33^	*U*^12^	*U*^13^	*U*^23^
N2	0.024 (2)	0.024 (2)	0.019 (2)	0.0044 (18)	0.0044 (17)	0.0013 (17)
C10	0.056 (4)	0.044 (4)	0.035 (3)	−0.010 (3)	0.015 (3)	−0.011 (3)
C11	0.076 (6)	0.040 (4)	0.086 (6)	−0.017 (4)	0.048 (5)	−0.026 (4)
C12	0.033 (4)	0.079 (6)	0.047 (4)	0.012 (4)	0.008 (3)	0.020 (4)
C13	0.026 (3)	0.130 (8)	0.027 (3)	−0.007 (4)	−0.004 (3)	0.005 (4)
C14	0.037 (3)	0.051 (4)	0.033 (3)	0.003 (3)	0.009 (3)	0.003 (3)
C15	0.026 (3)	0.042 (4)	0.034 (3)	0.000 (2)	0.003 (2)	−0.008 (3)
C16	0.047 (4)	0.029 (3)	0.052 (4)	−0.001 (3)	0.003 (3)	−0.004 (3)
C17	0.045 (4)	0.034 (3)	0.049 (4)	−0.007 (3)	0.007 (3)	0.005 (3)
Pt1	0.01677 (10)	0.01604 (10)	0.01721 (10)	0.00084 (7)	0.00130 (7)	0.00005 (7)
Br1	0.0303 (3)	0.0270 (3)	0.0244 (2)	−0.0012 (2)	−0.0021 (2)	−0.0027 (2)
Br2	0.0295 (3)	0.0267 (3)	0.0266 (3)	0.0050 (2)	0.0015 (2)	−0.0004 (2)
Br3	0.0262 (3)	0.0286 (3)	0.0303 (3)	0.0029 (2)	−0.0035 (2)	−0.0062 (2)
S1	0.0177 (6)	0.0245 (6)	0.0275 (6)	0.0036 (5)	0.0004 (5)	−0.0025 (5)
O1	0.047 (2)	0.022 (2)	0.032 (2)	0.0026 (18)	0.0023 (18)	0.0078 (16)
N1	0.019 (2)	0.018 (2)	0.0171 (19)	−0.0008 (16)	−0.0012 (16)	−0.0010 (16)
C1	0.018 (2)	0.022 (3)	0.023 (2)	0.0007 (19)	−0.0009 (19)	−0.003 (2)
C2	0.024 (2)	0.013 (2)	0.020 (2)	0.0017 (19)	0.0000 (19)	−0.0010 (18)
C3	0.021 (2)	0.020 (3)	0.026 (2)	0.001 (2)	0.004 (2)	−0.002 (2)
C4	0.028 (3)	0.024 (3)	0.025 (3)	−0.001 (2)	0.007 (2)	0.001 (2)
C5	0.041 (3)	0.016 (2)	0.021 (2)	0.000 (2)	0.000 (2)	−0.0010 (19)
C6	0.033 (3)	0.017 (2)	0.025 (2)	0.005 (2)	−0.004 (2)	−0.002 (2)
C7	0.021 (2)	0.018 (2)	0.022 (2)	0.0006 (19)	−0.0022 (19)	−0.0056 (19)
C8	0.024 (3)	0.027 (3)	0.033 (3)	−0.001 (2)	0.008 (2)	0.000 (2)
C9	0.053 (4)	0.025 (3)	0.037 (3)	−0.001 (3)	−0.011 (3)	0.007 (2)

### (2) Tetraethylammonium tribromido(6-methoxy-2-methyl-1,3-benzothiazole-κN)platinate(II) Geometric parameters (Å, º)

**Table:**

N2—C10	1.511 (7)	C17—H17C	0.9800
N2—C12	1.523 (8)	Pt1—Br1	2.4352 (5)
N2—C14	1.521 (7)	Pt1—Br2	2.4241 (6)
N2—C16	1.491 (8)	Pt1—Br3	2.4309 (5)
C10—H10A	0.9900	Pt1—N1	2.025 (4)
C10—H10B	0.9900	S1—C1	1.730 (5)
C10—C11	1.474 (10)	S1—C7	1.743 (5)
C11—H11A	0.9800	O1—C5	1.379 (6)
C11—H11B	0.9800	O1—C9	1.427 (7)
C11—H11C	0.9800	N1—C1	1.309 (6)
C12—H12A	0.9900	N1—C2	1.396 (6)
C12—H12B	0.9900	C1—C8	1.483 (7)
C12—C13	1.464 (11)	C2—C3	1.405 (7)
C13—H13A	0.9800	C2—C7	1.383 (7)
C13—H13B	0.9800	C3—H3	0.9500
C13—H13C	0.9800	C3—C4	1.377 (7)
C14—H14A	0.9900	C4—H4	0.9500
C14—H14B	0.9900	C4—C5	1.396 (8)
C14—C15	1.513 (8)	C5—C6	1.386 (8)
C15—H15A	0.9800	C6—H6	0.9500
C15—H15B	0.9800	C6—C7	1.404 (7)
C15—H15C	0.9800	C8—H8A	0.9800
C16—H16A	0.9900	C8—H8B	0.9800
C16—H16B	0.9900	C8—H8C	0.9800
C16—C17	1.517 (9)	C9—H9A	0.9800
C17—H17A	0.9800	C9—H9B	0.9800
C17—H17B	0.9800	C9—H9C	0.9800
			
C10—N2—C12	108.5 (5)	C16—C17—H17B	109.5
C10—N2—C14	111.4 (5)	C16—C17—H17C	109.5
C14—N2—C12	107.4 (4)	H17A—C17—H17B	109.5
C16—N2—C10	109.6 (5)	H17A—C17—H17C	109.5
C16—N2—C12	110.1 (5)	H17B—C17—H17C	109.5
C16—N2—C14	109.8 (5)	Br2—Pt1—Br1	91.171 (19)
N2—C10—H10A	107.9	Br2—Pt1—Br3	92.507 (19)
N2—C10—H10B	107.9	Br3—Pt1—Br1	176.30 (2)
H10A—C10—H10B	107.2	N1—Pt1—Br1	87.04 (11)
C11—C10—N2	117.8 (6)	N1—Pt1—Br2	177.41 (11)
C11—C10—H10A	107.9	N1—Pt1—Br3	89.29 (11)
C11—C10—H10B	107.9	C1—S1—C7	89.9 (2)
C10—C11—H11A	109.5	C5—O1—C9	116.3 (5)
C10—C11—H11B	109.5	C1—N1—Pt1	124.7 (3)
C10—C11—H11C	109.5	C1—N1—C2	112.6 (4)
H11A—C11—H11B	109.5	C2—N1—Pt1	122.1 (3)
H11A—C11—H11C	109.5	N1—C1—S1	114.0 (4)
H11B—C11—H11C	109.5	N1—C1—C8	124.2 (5)
N2—C12—H12A	108.0	C8—C1—S1	121.8 (4)
N2—C12—H12B	108.0	N1—C2—C3	126.5 (4)
H12A—C12—H12B	107.3	C7—C2—N1	113.5 (4)
C13—C12—N2	117.1 (6)	C7—C2—C3	120.0 (5)
C13—C12—H12A	108.0	C2—C3—H3	120.9
C13—C12—H12B	108.0	C4—C3—C2	118.2 (5)
C12—C13—H13A	109.5	C4—C3—H3	120.9
C12—C13—H13B	109.5	C3—C4—H4	119.4
C12—C13—H13C	109.5	C3—C4—C5	121.2 (5)
H13A—C13—H13B	109.5	C5—C4—H4	119.4
H13A—C13—H13C	109.5	O1—C5—C4	115.7 (5)
H13B—C13—H13C	109.5	O1—C5—C6	122.6 (5)
N2—C14—H14A	108.7	C6—C5—C4	121.7 (5)
N2—C14—H14B	108.7	C5—C6—H6	121.8
H14A—C14—H14B	107.6	C5—C6—C7	116.5 (5)
C15—C14—N2	114.3 (5)	C7—C6—H6	121.8
C15—C14—H14A	108.7	C2—C7—S1	109.9 (4)
C15—C14—H14B	108.7	C2—C7—C6	122.4 (5)
C14—C15—H15A	109.5	C6—C7—S1	127.7 (4)
C14—C15—H15B	109.5	C1—C8—H8A	109.5
C14—C15—H15C	109.5	C1—C8—H8B	109.5
H15A—C15—H15B	109.5	C1—C8—H8C	109.5
H15A—C15—H15C	109.5	H8A—C8—H8B	109.5
H15B—C15—H15C	109.5	H8A—C8—H8C	109.5
N2—C16—H16A	108.4	H8B—C8—H8C	109.5
N2—C16—H16B	108.4	O1—C9—H9A	109.5
N2—C16—C17	115.4 (5)	O1—C9—H9B	109.5
H16A—C16—H16B	107.5	O1—C9—H9C	109.5
C17—C16—H16A	108.4	H9A—C9—H9B	109.5
C17—C16—H16B	108.4	H9A—C9—H9C	109.5
C16—C17—H17A	109.5	H9B—C9—H9C	109.5

### (3) Tetraethylammonium tribromido(2,5,6-trimethyl-1,3-benzothiazole-κN)platinate(II) Crystal data

**Table:**

(C_8_H_20_N)[PtBr_3_(C_10_H_11_NS)]	*F*(000) = 1408
*M**_r_* = 742.33	*D*_x_ = 2.151 Mg m^−^^3^
Monoclinic, *P*2_1_/*n*	Mo *K*α radiation, λ = 0.71073 Å
*a* = 7.9742 (4) Å	Cell parameters from 5770 reflections
*b* = 30.2807 (14) Å	θ = 2.3–26.4°
*c* = 9.6427 (5) Å	µ = 11.45 mm^−^^1^
β = 100.151 (3)°	*T* = 100 K
*V* = 2291.9 (2) Å^3^	Block, red
*Z* = 4	0.5 × 0.36 × 0.25 mm

### (3) Tetraethylammonium tribromido(2,5,6-trimethyl-1,3-benzothiazole-κN)platinate(II) Data collection

**Table:**

Bruker APEXII CCD diffractometer	4692 independent reflections
Radiation source: Micro Focus Rotating Anode, Bruker TXS	4120 reflections with *I* > 2σ(*I*)
Double Bounce Multilayer Mirrors monochromator	*R*_int_ = 0.048
Detector resolution: 7.9 pixels mm^-1^	θ_max_ = 26.5°, θ_min_ = 2.3°
φ and ω scans	*h* = −9→5
Absorption correction: multi-scan (*SADABS*; Bruker, 2014)	*k* = −37→31
*T*_min_ = 0.003, *T*_max_ = 0.028	*l* = −10→12
10729 measured reflections	

### (3) Tetraethylammonium tribromido(2,5,6-trimethyl-1,3-benzothiazole-κN)platinate(II) Refinement

**Table:**

Refinement on *F*^2^	0 restraints
Least-squares matrix: full	Hydrogen site location: inferred from neighbouring sites
*R*[*F*^2^ > 2σ(*F*^2^)] = 0.039	H-atom parameters constrained
*wR*(*F*^2^) = 0.106	*w* = 1/[σ^2^(*F*_o_^2^) + (0.056*P*)^2^ + 1.6623*P*] where *P* = (*F*_o_^2^ + 2*F*_c_^2^)/3
*S* = 1.05	(Δ/σ)_max_ = 0.002
4692 reflections	Δρ_max_ = 1.88 e Å^−^^3^
266 parameters	Δρ_min_ = −1.02 e Å^−^^3^

### (3) Tetraethylammonium tribromido(2,5,6-trimethyl-1,3-benzothiazole-κN)platinate(II) Special details

**Table:**

Geometry. All e.s.d.'s (except the e.s.d. in the dihedral angle between two l.s. planes) are estimated using the full covariance matrix. The cell e.s.d.'s are taken into account individually in the estimation of e.s.d.'s in distances, angles and torsion angles; correlations between e.s.d.'s in cell parameters are only used when they are defined by crystal symmetry. An approximate (isotropic) treatment of cell e.s.d.'s is used for estimating e.s.d.'s involving l.s. planes.

### (3) Tetraethylammonium tribromido(2,5,6-trimethyl-1,3-benzothiazole-κN)platinate(II) Fractional atomic coordinates and isotropic or equivalent isotropic displacement parameters (Å^2^)

**Table:**

	*x*	*y*	*z*	*U*_iso_*/*U*_eq_	Occ. (<1)
N2	0.5342 (7)	0.18676 (17)	0.5028 (5)	0.0281 (11)	
C12	0.8178 (11)	0.1847 (4)	0.6760 (9)	0.060 (2)	
H12A	0.8673	0.1911	0.7743	0.089*	0.566 (9)
H12B	0.8346	0.1535	0.6560	0.089*	0.566 (9)
H12C	0.8736	0.2030	0.6137	0.089*	0.566 (9)
H12D	0.9301	0.1706	0.6901	0.089*	0.434 (9)
H12E	0.8313	0.2168	0.6706	0.089*	0.434 (9)
H12F	0.7606	0.1775	0.7551	0.089*	0.434 (9)
C14	0.4260 (12)	0.1207 (3)	0.3440 (10)	0.057 (2)	
H14A	0.4285	0.0884	0.3448	0.086*	0.566 (9)
H14B	0.3076	0.1309	0.3310	0.086*	0.566 (9)
H14C	0.4792	0.1316	0.2665	0.086*	0.566 (9)
H14D	0.3786	0.1145	0.2451	0.086*	0.434 (9)
H14E	0.5321	0.1041	0.3718	0.086*	0.434 (9)
H14F	0.3439	0.1119	0.4033	0.086*	0.434 (9)
C16	0.2437 (10)	0.1856 (2)	0.5783 (8)	0.0404 (17)	
H16A	0.1334	0.2008	0.5672	0.061*	0.566 (9)
H16B	0.2262	0.1549	0.5468	0.061*	0.566 (9)
H16C	0.2981	0.1862	0.6777	0.061*	0.566 (9)
H16D	0.1836	0.1759	0.6532	0.061*	0.434 (9)
H16E	0.2345	0.2178	0.5684	0.061*	0.434 (9)
H16F	0.1925	0.1716	0.4893	0.061*	0.434 (9)
C18	0.6616 (12)	0.2550 (3)	0.4092 (10)	0.054 (2)	
H18A	0.7199	0.2649	0.3334	0.081*	0.566 (9)
H18B	0.5547	0.2713	0.4045	0.081*	0.566 (9)
H18C	0.7348	0.2603	0.5004	0.081*	0.566 (9)
H18D	0.6529	0.2872	0.4073	0.081*	0.434 (9)
H18E	0.7769	0.2463	0.4540	0.081*	0.434 (9)
H18F	0.6378	0.2435	0.3126	0.081*	0.434 (9)
C11	0.6256 (17)	0.1951 (4)	0.6507 (12)	0.033 (3)	0.566 (9)
H11A	0.5715	0.1771	0.7162	0.040*	0.566 (9)
H11B	0.6104	0.2266	0.6739	0.040*	0.566 (9)
C13	0.5186 (18)	0.1373 (4)	0.4765 (14)	0.037 (3)	0.566 (9)
H13A	0.6356	0.1252	0.4883	0.044*	0.566 (9)
H13B	0.4650	0.1245	0.5524	0.044*	0.566 (9)
C15	0.3598 (15)	0.2095 (4)	0.4884 (12)	0.032 (3)	0.566 (9)
H15A	0.3039	0.2093	0.3882	0.038*	0.566 (9)
H15B	0.3758	0.2406	0.5193	0.038*	0.566 (9)
C17A	0.6257 (15)	0.2086 (5)	0.3936 (13)	0.040 (3)	0.566 (9)
H17A	0.7349	0.1930	0.3950	0.048*	0.566 (9)
H17B	0.5555	0.2039	0.2993	0.048*	0.566 (9)
C11A	0.716 (2)	0.1689 (6)	0.546 (2)	0.044 (5)	0.434 (9)
H11C	0.7089	0.1363	0.5527	0.053*	0.434 (9)
H11D	0.7792	0.1755	0.4687	0.053*	0.434 (9)
C13A	0.460 (2)	0.1664 (5)	0.3608 (15)	0.033 (4)	0.434 (9)
H13C	0.3509	0.1819	0.3266	0.040*	0.434 (9)
H13D	0.5377	0.1742	0.2953	0.040*	0.434 (9)
C15A	0.435 (2)	0.1723 (6)	0.6165 (14)	0.031 (4)	0.434 (9)
H15C	0.4863	0.1860	0.7073	0.038*	0.434 (9)
H15D	0.4442	0.1398	0.6281	0.038*	0.434 (9)
C17	0.535 (2)	0.2363 (5)	0.4914 (17)	0.033 (4)	0.434 (9)
H17C	0.4195	0.2461	0.4466	0.040*	0.434 (9)
H17D	0.5582	0.2489	0.5876	0.040*	0.434 (9)
Pt1	0.28820 (3)	0.36639 (2)	0.54603 (2)	0.02321 (10)	
Br1	0.11056 (8)	0.41268 (2)	0.37347 (7)	0.03333 (17)	
Br2	0.08097 (9)	0.30729 (2)	0.50744 (7)	0.03324 (17)	
Br3	0.47121 (9)	0.32318 (2)	0.72302 (8)	0.04046 (19)	
S1	0.7243 (2)	0.46490 (5)	0.58781 (16)	0.0279 (3)	
N1	0.4604 (6)	0.41615 (16)	0.5825 (5)	0.0239 (10)	
C1	0.6036 (8)	0.4185 (2)	0.5354 (6)	0.0271 (13)	
C2	0.4361 (8)	0.4523 (2)	0.6672 (6)	0.0258 (13)	
C3	0.2953 (8)	0.4599 (2)	0.7319 (6)	0.0263 (13)	
H3	0.2031	0.4396	0.7202	0.032*	
C4	0.2917 (8)	0.4974 (2)	0.8136 (6)	0.0285 (13)	
C5	0.4298 (9)	0.5269 (2)	0.8313 (7)	0.0319 (14)	
C6	0.5667 (9)	0.5201 (2)	0.7669 (6)	0.0294 (14)	
H6	0.6578	0.5408	0.7782	0.035*	
C7	0.5713 (8)	0.4824 (2)	0.6840 (6)	0.0263 (13)	
C8	0.6633 (9)	0.3844 (2)	0.4455 (7)	0.0342 (15)	
H8A	0.5737	0.3784	0.3642	0.051*	
H8B	0.6900	0.3572	0.5001	0.051*	
H8C	0.7657	0.3949	0.4126	0.051*	
C9	0.1406 (9)	0.5053 (2)	0.8848 (7)	0.0346 (15)	
H9A	0.0846	0.5330	0.8503	0.052*	
H9B	0.1792	0.5073	0.9869	0.052*	
H9C	0.0599	0.4808	0.8636	0.052*	
C10	0.4240 (10)	0.5680 (2)	0.9223 (8)	0.0391 (16)	
H10A	0.3289	0.5869	0.8798	0.059*	
H10B	0.5311	0.5844	0.9286	0.059*	
H10C	0.4084	0.5591	1.0170	0.059*	

### (3) Tetraethylammonium tribromido(2,5,6-trimethyl-1,3-benzothiazole-κN)platinate(II) Atomic displacement parameters (Å^2^)

**Table:**

	*U*^11^	*U*^22^	*U*^33^	*U*^12^	*U*^13^	*U*^23^
N2	0.028 (3)	0.026 (3)	0.031 (3)	0.001 (2)	0.007 (2)	−0.001 (2)
C12	0.036 (4)	0.093 (7)	0.047 (5)	0.003 (4)	−0.002 (4)	0.015 (5)
C14	0.053 (5)	0.052 (5)	0.072 (6)	−0.005 (4)	0.026 (5)	−0.035 (5)
C16	0.038 (4)	0.037 (4)	0.051 (4)	0.000 (3)	0.023 (3)	−0.005 (3)
C18	0.061 (5)	0.045 (5)	0.060 (5)	−0.009 (4)	0.021 (4)	0.014 (4)
C11	0.045 (7)	0.027 (6)	0.027 (5)	−0.004 (5)	0.004 (5)	−0.001 (5)
C13	0.038 (7)	0.028 (6)	0.043 (7)	0.009 (5)	0.004 (6)	−0.008 (5)
C15	0.037 (6)	0.023 (6)	0.034 (6)	0.003 (5)	0.007 (5)	−0.002 (5)
C17A	0.024 (6)	0.063 (9)	0.034 (6)	0.000 (6)	0.010 (5)	0.003 (6)
C11A	0.035 (9)	0.044 (10)	0.058 (11)	0.017 (8)	0.020 (8)	0.022 (8)
C13A	0.049 (9)	0.032 (8)	0.023 (7)	−0.013 (7)	0.014 (7)	−0.013 (6)
C15A	0.032 (8)	0.044 (9)	0.020 (7)	0.000 (7)	0.009 (6)	0.006 (6)
C17	0.037 (8)	0.031 (8)	0.037 (8)	−0.009 (7)	0.018 (7)	0.001 (6)
Pt1	0.02518 (15)	0.01967 (15)	0.02593 (15)	−0.00120 (8)	0.00770 (10)	0.00056 (8)
Br1	0.0312 (3)	0.0284 (3)	0.0401 (4)	−0.0015 (3)	0.0056 (3)	0.0073 (3)
Br2	0.0415 (4)	0.0266 (3)	0.0321 (3)	−0.0074 (3)	0.0077 (3)	0.0004 (2)
Br3	0.0389 (4)	0.0386 (4)	0.0420 (4)	0.0009 (3)	0.0019 (3)	0.0113 (3)
S1	0.0268 (7)	0.0263 (8)	0.0320 (8)	−0.0029 (6)	0.0091 (6)	0.0027 (6)
N1	0.026 (3)	0.025 (3)	0.021 (2)	0.001 (2)	0.006 (2)	0.002 (2)
C1	0.030 (3)	0.025 (3)	0.026 (3)	−0.004 (2)	0.006 (3)	0.000 (2)
C2	0.023 (3)	0.027 (3)	0.028 (3)	−0.001 (2)	0.007 (2)	0.006 (2)
C3	0.028 (3)	0.022 (3)	0.030 (3)	−0.003 (2)	0.007 (3)	0.002 (2)
C4	0.033 (3)	0.023 (3)	0.031 (3)	0.004 (3)	0.009 (3)	−0.001 (3)
C5	0.046 (4)	0.025 (3)	0.025 (3)	0.004 (3)	0.005 (3)	0.000 (2)
C6	0.037 (4)	0.021 (3)	0.030 (3)	−0.005 (3)	0.005 (3)	0.005 (2)
C7	0.032 (3)	0.022 (3)	0.026 (3)	0.002 (2)	0.007 (3)	0.007 (2)
C8	0.030 (3)	0.037 (4)	0.039 (4)	0.001 (3)	0.013 (3)	−0.005 (3)
C9	0.045 (4)	0.027 (3)	0.034 (3)	0.001 (3)	0.012 (3)	0.001 (3)
C10	0.050 (4)	0.020 (3)	0.048 (4)	−0.004 (3)	0.013 (3)	−0.004 (3)

### (3) Tetraethylammonium tribromido(2,5,6-trimethyl-1,3-benzothiazole-κN)platinate(II) Geometric parameters (Å, º)

**Table:**

N2—C11	1.504 (12)	C13—H13A	0.9900
N2—C13	1.520 (12)	C13—H13B	0.9900
N2—C15	1.535 (13)	C15—H15A	0.9900
N2—C17A	1.533 (13)	C15—H15B	0.9900
N2—C11A	1.537 (16)	C17A—H17A	0.9900
N2—C13A	1.523 (14)	C17A—H17B	0.9900
N2—C15A	1.523 (15)	C11A—H11C	0.9900
N2—C17	1.505 (16)	C11A—H11D	0.9900
C12—H12A	0.9800	C13A—H13C	0.9900
C12—H12B	0.9800	C13A—H13D	0.9900
C12—H12C	0.9800	C15A—H15C	0.9900
C12—H12D	0.9800	C15A—H15D	0.9900
C12—H12E	0.9800	C17—H17C	0.9900
C12—H12F	0.9800	C17—H17D	0.9900
C12—C11	1.542 (15)	Pt1—Br1	2.4309 (7)
C12—C11A	1.45 (2)	Pt1—Br2	2.4198 (7)
C14—H14A	0.9800	Pt1—Br3	2.4240 (7)
C14—H14B	0.9800	Pt1—N1	2.027 (5)
C14—H14C	0.9800	S1—C1	1.727 (6)
C14—H14D	0.9800	S1—C7	1.739 (7)
C14—H14E	0.9800	N1—C1	1.303 (8)
C14—H14F	0.9800	N1—C2	1.401 (8)
C14—C13	1.449 (15)	C1—C8	1.482 (9)
C14—C13A	1.415 (16)	C2—C3	1.397 (9)
C16—H16A	0.9800	C2—C7	1.398 (9)
C16—H16B	0.9800	C3—H3	0.9500
C16—H16C	0.9800	C3—C4	1.385 (9)
C16—H16D	0.9800	C4—C5	1.404 (9)
C16—H16E	0.9800	C4—C9	1.507 (9)
C16—H16F	0.9800	C5—C6	1.363 (10)
C16—C15	1.553 (14)	C5—C10	1.528 (9)
C16—C15A	1.561 (17)	C6—H6	0.9500
C18—H18A	0.9800	C6—C7	1.400 (9)
C18—H18B	0.9800	C8—H8A	0.9800
C18—H18C	0.9800	C8—H8B	0.9800
C18—H18D	0.9800	C8—H8C	0.9800
C18—H18E	0.9800	C9—H9A	0.9800
C18—H18F	0.9800	C9—H9B	0.9800
C18—C17A	1.435 (17)	C9—H9C	0.9800
C18—C17	1.500 (16)	C10—H10A	0.9800
C11—H11A	0.9900	C10—H10B	0.9800
C11—H11B	0.9900	C10—H10C	0.9800
			
C11—N2—C13	109.7 (7)	N2—C17A—H17B	107.9
C11—N2—C15	106.9 (8)	C18—C17A—N2	117.4 (10)
C11—N2—C17A	111.6 (8)	C18—C17A—H17A	107.9
C13—N2—C15	112.3 (8)	C18—C17A—H17B	107.9
C13—N2—C17A	110.2 (8)	H17A—C17A—H17B	107.2
C17A—N2—C15	106.1 (7)	N2—C11A—H11C	107.7
C13A—N2—C11A	107.5 (11)	N2—C11A—H11D	107.7
C13A—N2—C15A	111.2 (9)	C12—C11A—N2	118.3 (13)
C15A—N2—C11A	106.7 (9)	C12—C11A—H11C	107.7
C17—N2—C11A	110.9 (10)	C12—C11A—H11D	107.7
C17—N2—C13A	110.0 (9)	H11C—C11A—H11D	107.1
C17—N2—C15A	110.4 (9)	N2—C13A—H13C	106.7
H12A—C12—H12B	109.5	N2—C13A—H13D	106.7
H12A—C12—H12C	109.5	C14—C13A—N2	122.3 (12)
H12B—C12—H12C	109.5	C14—C13A—H13C	106.7
H12D—C12—H12E	109.5	C14—C13A—H13D	106.7
H12D—C12—H12F	109.5	H13C—C13A—H13D	106.6
H12E—C12—H12F	109.5	N2—C15A—C16	111.4 (9)
C11—C12—H12A	109.5	N2—C15A—H15C	109.4
C11—C12—H12B	109.5	N2—C15A—H15D	109.4
C11—C12—H12C	109.5	C16—C15A—H15C	109.4
C11A—C12—H12D	109.5	C16—C15A—H15D	109.4
C11A—C12—H12E	109.5	H15C—C15A—H15D	108.0
C11A—C12—H12F	109.5	N2—C17—H17C	108.5
H14A—C14—H14B	109.5	N2—C17—H17D	108.5
H14A—C14—H14C	109.5	C18—C17—N2	115.2 (11)
H14B—C14—H14C	109.5	C18—C17—H17C	108.5
H14D—C14—H14E	109.5	C18—C17—H17D	108.5
H14D—C14—H14F	109.5	H17C—C17—H17D	107.5
H14E—C14—H14F	109.5	Br2—Pt1—Br1	91.23 (2)
C13—C14—H14A	109.5	Br2—Pt1—Br3	91.10 (2)
C13—C14—H14B	109.5	Br3—Pt1—Br1	177.45 (3)
C13—C14—H14C	109.5	N1—Pt1—Br1	89.16 (14)
C13A—C14—H14D	109.5	N1—Pt1—Br2	178.76 (14)
C13A—C14—H14E	109.5	N1—Pt1—Br3	88.50 (14)
C13A—C14—H14F	109.5	C1—S1—C7	89.8 (3)
H16A—C16—H16B	109.5	C1—N1—Pt1	126.1 (4)
H16A—C16—H16C	109.5	C1—N1—C2	112.3 (5)
H16B—C16—H16C	109.5	C2—N1—Pt1	121.6 (4)
H16D—C16—H16E	109.5	N1—C1—S1	114.9 (5)
H16D—C16—H16F	109.5	N1—C1—C8	123.9 (6)
H16E—C16—H16F	109.5	C8—C1—S1	121.2 (5)
C15—C16—H16A	109.5	C3—C2—N1	126.5 (6)
C15—C16—H16B	109.5	C3—C2—C7	120.4 (6)
C15—C16—H16C	109.5	C7—C2—N1	113.1 (5)
C15A—C16—H16D	109.5	C2—C3—H3	120.4
C15A—C16—H16E	109.5	C4—C3—C2	119.2 (6)
C15A—C16—H16F	109.5	C4—C3—H3	120.4
H18A—C18—H18B	109.5	C3—C4—C5	119.8 (6)
H18A—C18—H18C	109.5	C3—C4—C9	119.1 (6)
H18B—C18—H18C	109.5	C5—C4—C9	121.1 (6)
H18D—C18—H18E	109.5	C4—C5—C10	119.0 (6)
H18D—C18—H18F	109.5	C6—C5—C4	121.5 (6)
H18E—C18—H18F	109.5	C6—C5—C10	119.5 (6)
C17A—C18—H18A	109.5	C5—C6—H6	120.4
C17A—C18—H18B	109.5	C5—C6—C7	119.1 (6)
C17A—C18—H18C	109.5	C7—C6—H6	120.4
C17—C18—H18D	109.5	C2—C7—S1	109.9 (5)
C17—C18—H18E	109.5	C2—C7—C6	120.0 (6)
C17—C18—H18F	109.5	C6—C7—S1	130.1 (5)
N2—C11—C12	114.7 (9)	C1—C8—H8A	109.5
N2—C11—H11A	108.6	C1—C8—H8B	109.5
N2—C11—H11B	108.6	C1—C8—H8C	109.5
C12—C11—H11A	108.6	H8A—C8—H8B	109.5
C12—C11—H11B	108.6	H8A—C8—H8C	109.5
H11A—C11—H11B	107.6	H8B—C8—H8C	109.5
N2—C13—H13A	107.3	C4—C9—H9A	109.5
N2—C13—H13B	107.3	C4—C9—H9B	109.5
C14—C13—N2	120.2 (10)	C4—C9—H9C	109.5
C14—C13—H13A	107.3	H9A—C9—H9B	109.5
C14—C13—H13B	107.3	H9A—C9—H9C	109.5
H13A—C13—H13B	106.9	H9B—C9—H9C	109.5
N2—C15—C16	111.2 (8)	C5—C10—H10A	109.5
N2—C15—H15A	109.4	C5—C10—H10B	109.5
N2—C15—H15B	109.4	C5—C10—H10C	109.5
C16—C15—H15A	109.4	H10A—C10—H10B	109.5
C16—C15—H15B	109.4	H10A—C10—H10C	109.5
H15A—C15—H15B	108.0	H10B—C10—H10C	109.5
N2—C17A—H17A	107.9		

### (4) Tetraethylammonium tribromido(2-methyl-5-nitro-1,3-benzothiazole-κN)platinate(II) Crystal data

**Table:**

(C_8_H_20_N)[PtBr_3_(C_8_H_6_N_2_O_2_S)]	*F*(000) = 1432
*M**_r_* = 759.28	*D*_x_ = 2.272 Mg m^−^^3^
Monoclinic, *P*2_1_/*n*	Mo *K*α radiation, λ = 0.71073 Å
*a* = 8.1170 (3) Å	Cell parameters from 9483 reflections
*b* = 29.2717 (12) Å	θ = 2.6–26.4°
*c* = 9.5102 (4) Å	µ = 11.83 mm^−^^1^
β = 100.720 (1)°	*T* = 100 K
*V* = 2220.17 (15) Å^3^	Block, bronze
*Z* = 4	0.32 × 0.3 × 0.25 mm

### (4) Tetraethylammonium tribromido(2-methyl-5-nitro-1,3-benzothiazole-κN)platinate(II) Data collection

**Table:**

Bruker APEXII CCD diffractometer	4550 independent reflections
Radiation source: Micro Focus Rotating Anode, Bruker TXS	4254 reflections with *I* > 2σ(*I*)
Double Bounce Multilayer Mirrors monochromator	*R*_int_ = 0.028
Detector resolution: 7.9 pixels mm^-1^	θ_max_ = 26.5°, θ_min_ = 2.3°
φ and ω scans	*h* = −10→10
Absorption correction: multi-scan (*SADABS*; Bruker, 2014)	*k* = −31→36
*T*_min_ = 0.020, *T*_max_ = 0.045	*l* = −11→7
15975 measured reflections	

### (4) Tetraethylammonium tribromido(2-methyl-5-nitro-1,3-benzothiazole-κN)platinate(II) Refinement

**Table:**

Refinement on *F*^2^	0 restraints
Least-squares matrix: full	Hydrogen site location: inferred from neighbouring sites
*R*[*F*^2^ > 2σ(*F*^2^)] = 0.029	H-atom parameters constrained
*wR*(*F*^2^) = 0.060	*w* = 1/[σ^2^(*F*_o_^2^) + (0.0044*P*)^2^ + 13.0832*P*] where *P* = (*F*_o_^2^ + 2*F*_c_^2^)/3
*S* = 1.18	(Δ/σ)_max_ = 0.002
4550 reflections	Δρ_max_ = 1.25 e Å^−^^3^
240 parameters	Δρ_min_ = −1.37 e Å^−^^3^

### (4) Tetraethylammonium tribromido(2-methyl-5-nitro-1,3-benzothiazole-κN)platinate(II) Special details

**Table:**

Geometry. All e.s.d.'s (except the e.s.d. in the dihedral angle between two l.s. planes) are estimated using the full covariance matrix. The cell e.s.d.'s are taken into account individually in the estimation of e.s.d.'s in distances, angles and torsion angles; correlations between e.s.d.'s in cell parameters are only used when they are defined by crystal symmetry. An approximate (isotropic) treatment of cell e.s.d.'s is used for estimating e.s.d.'s involving l.s. planes.

### (4) Tetraethylammonium tribromido(2-methyl-5-nitro-1,3-benzothiazole-κN)platinate(II) Fractional atomic coordinates and isotropic or equivalent isotropic displacement parameters (Å^2^)

**Table:**

	*x*	*y*	*z*	*U*_iso_*/*U*_eq_	
N3	0.5548 (5)	0.31569 (15)	0.5135 (4)	0.0187 (9)	
C9	0.3795 (6)	0.30025 (19)	0.5223 (6)	0.0247 (12)	
H9A	0.3850	0.2685	0.5585	0.030*	
H9B	0.3112	0.3000	0.4245	0.030*	
C10	0.2911 (7)	0.3294 (2)	0.6170 (7)	0.0320 (13)	
H10A	0.2786	0.3606	0.5790	0.048*	
H10B	0.3575	0.3300	0.7143	0.048*	
H10C	0.1802	0.3165	0.6191	0.048*	
C11	0.5608 (7)	0.36508 (18)	0.4693 (6)	0.0257 (12)	
H11A	0.6776	0.3727	0.4612	0.031*	
H11B	0.5304	0.3844	0.5459	0.031*	
C12	0.4466 (7)	0.3771 (2)	0.3292 (6)	0.0281 (12)	
H12A	0.4815	0.3601	0.2510	0.042*	
H12B	0.4534	0.4099	0.3115	0.042*	
H12C	0.3308	0.3689	0.3347	0.042*	
C13	0.6205 (7)	0.28611 (19)	0.4041 (6)	0.0262 (12)	
H13A	0.5380	0.2869	0.3133	0.031*	
H13B	0.7258	0.2998	0.3853	0.031*	
C14	0.6543 (8)	0.2369 (2)	0.4470 (7)	0.0323 (13)	
H14A	0.6871	0.2201	0.3675	0.048*	
H14B	0.5527	0.2232	0.4707	0.048*	
H14C	0.7452	0.2354	0.5307	0.048*	
C15	0.6624 (7)	0.3097 (2)	0.6607 (6)	0.0266 (12)	
H15A	0.6503	0.2780	0.6929	0.032*	
H15B	0.6197	0.3303	0.7284	0.032*	
C16	0.8519 (7)	0.3199 (3)	0.6674 (7)	0.0373 (15)	
H16A	0.8974	0.2984	0.6054	0.056*	
H16B	0.9122	0.3164	0.7661	0.056*	
H16C	0.8655	0.3512	0.6348	0.056*	
Pt1	0.19959 (2)	0.63310 (2)	0.93287 (2)	0.01542 (6)	
Br1	0.01278 (6)	0.67668 (2)	0.75173 (6)	0.02534 (12)	
Br2	0.39391 (7)	0.69652 (2)	0.97693 (6)	0.02338 (12)	
Br3	0.38687 (6)	0.58556 (2)	1.10249 (6)	0.02324 (12)	
S1	−0.23297 (16)	0.53228 (4)	0.89580 (14)	0.0211 (3)	
O1	0.4596 (5)	0.50291 (14)	0.6504 (4)	0.0304 (9)	
O2	0.3206 (5)	0.44662 (14)	0.5385 (5)	0.0343 (10)	
N1	0.0320 (5)	0.58046 (14)	0.8995 (4)	0.0168 (8)	
N2	0.3324 (6)	0.48001 (15)	0.6180 (5)	0.0247 (10)	
C1	−0.1103 (6)	0.57965 (17)	0.9469 (6)	0.0187 (10)	
C2	0.0503 (6)	0.54253 (17)	0.8172 (5)	0.0187 (10)	
C3	0.1902 (6)	0.53230 (17)	0.7554 (5)	0.0192 (10)	
H3	0.2846	0.5520	0.7661	0.023*	
C4	0.1839 (7)	0.49242 (17)	0.6787 (6)	0.0213 (11)	
C5	0.0465 (7)	0.46309 (19)	0.6567 (6)	0.0263 (12)	
H5	0.0473	0.4365	0.5994	0.032*	
C6	−0.0899 (7)	0.47253 (18)	0.7177 (6)	0.0245 (12)	
H6	−0.1844	0.4528	0.7047	0.029*	
C7	−0.0852 (6)	0.51233 (18)	0.7997 (6)	0.0212 (11)	
C8	−0.1675 (7)	0.61580 (18)	1.0364 (6)	0.0239 (11)	
H8A	−0.1978	0.6433	0.9784	0.036*	
H8B	−0.0770	0.6231	1.1168	0.036*	
H8C	−0.2655	0.6049	1.0732	0.036*	

### (4) Tetraethylammonium tribromido(2-methyl-5-nitro-1,3-benzothiazole-κN)platinate(II) Atomic displacement parameters (Å^2^)

**Table:**

	*U*^11^	*U*^22^	*U*^33^	*U*^12^	*U*^13^	*U*^23^
N3	0.018 (2)	0.024 (2)	0.014 (2)	0.0008 (17)	0.0023 (17)	0.0010 (17)
C9	0.020 (3)	0.027 (3)	0.027 (3)	−0.003 (2)	0.006 (2)	0.004 (2)
C10	0.032 (3)	0.036 (3)	0.031 (3)	0.002 (3)	0.013 (3)	0.005 (3)
C11	0.025 (3)	0.024 (3)	0.029 (3)	−0.004 (2)	0.007 (2)	0.002 (2)
C12	0.035 (3)	0.028 (3)	0.022 (3)	0.005 (2)	0.009 (2)	0.007 (2)
C13	0.030 (3)	0.032 (3)	0.020 (3)	−0.002 (2)	0.011 (2)	−0.004 (2)
C14	0.034 (3)	0.031 (3)	0.033 (3)	0.011 (2)	0.008 (3)	−0.004 (3)
C15	0.025 (3)	0.033 (3)	0.022 (3)	0.000 (2)	0.002 (2)	−0.003 (2)
C16	0.018 (3)	0.064 (4)	0.026 (3)	0.004 (3)	−0.006 (2)	−0.011 (3)
Pt1	0.01462 (10)	0.01661 (9)	0.01517 (10)	−0.00278 (7)	0.00313 (7)	−0.00054 (7)
Br1	0.0203 (3)	0.0302 (3)	0.0244 (3)	−0.0025 (2)	0.0012 (2)	0.0072 (2)
Br2	0.0250 (3)	0.0238 (3)	0.0213 (3)	−0.0058 (2)	0.0043 (2)	−0.0007 (2)
Br3	0.0194 (2)	0.0225 (3)	0.0263 (3)	−0.0023 (2)	−0.0001 (2)	0.0038 (2)
S1	0.0183 (6)	0.0210 (6)	0.0235 (7)	−0.0059 (5)	0.0029 (5)	0.0014 (5)
O1	0.026 (2)	0.034 (2)	0.032 (2)	−0.0023 (17)	0.0070 (18)	−0.0038 (18)
O2	0.046 (3)	0.024 (2)	0.037 (2)	−0.0025 (18)	0.018 (2)	−0.0089 (18)
N1	0.014 (2)	0.019 (2)	0.016 (2)	−0.0005 (16)	0.0018 (17)	0.0022 (17)
N2	0.035 (3)	0.019 (2)	0.021 (2)	0.002 (2)	0.008 (2)	0.0032 (19)
C1	0.017 (2)	0.018 (2)	0.022 (3)	−0.0015 (19)	0.004 (2)	0.005 (2)
C2	0.023 (3)	0.017 (2)	0.014 (2)	−0.003 (2)	−0.001 (2)	0.0027 (19)
C3	0.017 (2)	0.021 (2)	0.018 (3)	−0.003 (2)	0.000 (2)	0.004 (2)
C4	0.026 (3)	0.019 (2)	0.018 (3)	0.000 (2)	0.002 (2)	0.004 (2)
C5	0.036 (3)	0.020 (3)	0.023 (3)	−0.006 (2)	0.005 (2)	−0.001 (2)
C6	0.026 (3)	0.023 (3)	0.022 (3)	−0.010 (2)	−0.001 (2)	0.000 (2)
C7	0.022 (3)	0.021 (3)	0.020 (3)	0.000 (2)	0.002 (2)	0.006 (2)
C8	0.020 (3)	0.022 (3)	0.030 (3)	−0.002 (2)	0.006 (2)	0.000 (2)

### (4) Tetraethylammonium tribromido(2-methyl-5-nitro-1,3-benzothiazole-κN)platinate(II) Geometric parameters (Å, º)

**Table:**

N3—C9	1.510 (6)	C16—H16B	0.9800
N3—C11	1.509 (7)	C16—H16C	0.9800
N3—C13	1.524 (6)	Pt1—Br1	2.4335 (6)
N3—C15	1.516 (7)	Pt1—Br2	2.4216 (5)
C9—H9A	0.9900	Pt1—Br3	2.4367 (5)
C9—H9B	0.9900	Pt1—N1	2.041 (4)
C9—C10	1.515 (8)	S1—C1	1.724 (5)
C10—H10A	0.9800	S1—C7	1.738 (5)
C10—H10B	0.9800	O1—N2	1.221 (6)
C10—H10C	0.9800	O2—N2	1.228 (6)
C11—H11A	0.9900	N1—C1	1.315 (6)
C11—H11B	0.9900	N1—C2	1.383 (6)
C11—C12	1.516 (8)	N2—C4	1.476 (7)
C12—H12A	0.9800	C1—C8	1.486 (7)
C12—H12B	0.9800	C2—C3	1.405 (7)
C12—H12C	0.9800	C2—C7	1.397 (7)
C13—H13A	0.9900	C3—H3	0.9500
C13—H13B	0.9900	C3—C4	1.372 (7)
C13—C14	1.509 (8)	C4—C5	1.392 (7)
C14—H14A	0.9800	C5—H5	0.9500
C14—H14B	0.9800	C5—C6	1.370 (8)
C14—H14C	0.9800	C6—H6	0.9500
C15—H15A	0.9900	C6—C7	1.399 (7)
C15—H15B	0.9900	C8—H8A	0.9800
C15—C16	1.557 (8)	C8—H8B	0.9800
C16—H16A	0.9800	C8—H8C	0.9800
			
C9—N3—C13	108.7 (4)	C16—C15—H15B	108.7
C9—N3—C15	107.5 (4)	C15—C16—H16A	109.5
C11—N3—C9	112.4 (4)	C15—C16—H16B	109.5
C11—N3—C13	108.7 (4)	C15—C16—H16C	109.5
C11—N3—C15	108.9 (4)	H16A—C16—H16B	109.5
C15—N3—C13	110.5 (4)	H16A—C16—H16C	109.5
N3—C9—H9A	108.6	H16B—C16—H16C	109.5
N3—C9—H9B	108.6	Br1—Pt1—Br3	176.23 (2)
N3—C9—C10	114.8 (5)	Br2—Pt1—Br1	91.183 (19)
H9A—C9—H9B	107.5	Br2—Pt1—Br3	90.989 (19)
C10—C9—H9A	108.6	N1—Pt1—Br1	88.64 (11)
C10—C9—H9B	108.6	N1—Pt1—Br2	178.40 (12)
C9—C10—H10A	109.5	N1—Pt1—Br3	89.28 (11)
C9—C10—H10B	109.5	C1—S1—C7	90.0 (2)
C9—C10—H10C	109.5	C1—N1—Pt1	124.2 (3)
H10A—C10—H10B	109.5	C1—N1—C2	111.9 (4)
H10A—C10—H10C	109.5	C2—N1—Pt1	123.8 (3)
H10B—C10—H10C	109.5	O1—N2—O2	123.9 (5)
N3—C11—H11A	108.6	O1—N2—C4	118.7 (4)
N3—C11—H11B	108.6	O2—N2—C4	117.4 (5)
N3—C11—C12	114.9 (5)	N1—C1—S1	114.6 (4)
H11A—C11—H11B	107.5	N1—C1—C8	124.8 (5)
C12—C11—H11A	108.6	C8—C1—S1	120.6 (4)
C12—C11—H11B	108.6	N1—C2—C3	126.0 (5)
C11—C12—H12A	109.5	N1—C2—C7	114.2 (4)
C11—C12—H12B	109.5	C7—C2—C3	119.7 (5)
C11—C12—H12C	109.5	C2—C3—H3	121.6
H12A—C12—H12B	109.5	C4—C3—C2	116.8 (5)
H12A—C12—H12C	109.5	C4—C3—H3	121.6
H12B—C12—H12C	109.5	C3—C4—N2	117.7 (5)
N3—C13—H13A	108.5	C3—C4—C5	123.6 (5)
N3—C13—H13B	108.5	C5—C4—N2	118.6 (5)
H13A—C13—H13B	107.5	C4—C5—H5	120.0
C14—C13—N3	115.3 (4)	C6—C5—C4	120.0 (5)
C14—C13—H13A	108.5	C6—C5—H5	120.0
C14—C13—H13B	108.5	C5—C6—H6	121.2
C13—C14—H14A	109.5	C5—C6—C7	117.6 (5)
C13—C14—H14B	109.5	C7—C6—H6	121.2
C13—C14—H14C	109.5	C2—C7—S1	109.3 (4)
H14A—C14—H14B	109.5	C2—C7—C6	122.2 (5)
H14A—C14—H14C	109.5	C6—C7—S1	128.5 (4)
H14B—C14—H14C	109.5	C1—C8—H8A	109.5
N3—C15—H15A	108.7	C1—C8—H8B	109.5
N3—C15—H15B	108.7	C1—C8—H8C	109.5
N3—C15—C16	114.3 (5)	H8A—C8—H8B	109.5
H15A—C15—H15B	107.6	H8A—C8—H8C	109.5
C16—C15—H15A	108.7	H8B—C8—H8C	109.5
			
C9—N3—C11—C12	−55.8 (6)	N1—C2—C7—S1	−2.1 (6)
C9—N3—C13—C14	−68.6 (6)	N1—C2—C7—C6	178.8 (5)
C9—N3—C15—C16	174.6 (5)	N2—C4—C5—C6	176.8 (5)
C11—N3—C9—C10	−54.2 (6)	C1—S1—C7—C2	1.9 (4)
C11—N3—C13—C14	168.7 (5)	C1—S1—C7—C6	−179.1 (5)
C11—N3—C15—C16	−63.3 (6)	C1—N1—C2—C3	−177.5 (5)
C13—N3—C9—C10	−174.6 (5)	C1—N1—C2—C7	1.1 (6)
C13—N3—C11—C12	64.6 (6)	C2—N1—C1—S1	0.4 (6)
C13—N3—C15—C16	56.0 (6)	C2—N1—C1—C8	−179.1 (5)
C15—N3—C9—C10	65.7 (6)	C2—C3—C4—N2	−177.5 (4)
C15—N3—C11—C12	−174.9 (4)	C2—C3—C4—C5	1.9 (8)
C15—N3—C13—C14	49.2 (6)	C3—C2—C7—S1	176.6 (4)
Pt1—N1—C1—S1	176.9 (2)	C3—C2—C7—C6	−2.4 (8)
Pt1—N1—C1—C8	−2.6 (7)	C3—C4—C5—C6	−2.6 (8)
Pt1—N1—C2—C3	6.0 (7)	C4—C5—C6—C7	0.7 (8)
Pt1—N1—C2—C7	−175.4 (3)	C5—C6—C7—S1	−177.1 (4)
O1—N2—C4—C3	7.5 (7)	C5—C6—C7—C2	1.7 (8)
O1—N2—C4—C5	−171.9 (5)	C7—S1—C1—N1	−1.4 (4)
O2—N2—C4—C3	−173.4 (5)	C7—S1—C1—C8	178.2 (5)
O2—N2—C4—C5	7.2 (7)	C7—C2—C3—C4	0.6 (7)
N1—C2—C3—C4	179.2 (5)		
